# Causal Exposures of Immune Cells in Neuromyelitis Optica Spectrum Disorders: A Mendelian Randomization Study and Flow Cytometry Analysis

**DOI:** 10.1002/brb3.71400

**Published:** 2026-04-16

**Authors:** Jingru Ren, Jianchun Wang, Zhenyu Niu, Jing Guo, Nan Zhang, Hongjun Hao, Feng Gao, Ran Liu, Zhaoxia Wang

**Affiliations:** ^1^ Department of Neurology Peking University First Hospital Beijing China

**Keywords:** aquaporin‐4 (AQP4), flow cytometry analysis, immune cells, Mendelian randomization, neuromyelitis optica spectrum disorders (NMOSD)

## Abstract

**Background:**

Neuromyelitis optica spectrum disorders (NMOSD) are an immune‐mediated inflammatory disease of the central nervous system (CNS) primarily characterized by anti‐aquaporin‐4 immunoglobulin G (AQP4‐IgG)‐mediated astrocyte injury, neuroinflammation, and demyelination. However, the relationship between the disease and the immune trait from a genetic perspective still requires further confirmation.

**Methods:**

This study used two‐sample Mendelian randomization (MR) to assess causal relationship of immune cells with NMOSD. A total of 731 immune phenotyping traits were considered as exposure factors. NMOSD group is defined as outcome factor, which single‐nucleotide polymorphisms (SNPs) obtained from the study of Karol Estrada et al. (*n* = 215 NMOSD cases). The disease group was subcategorized into three groups based on the presence of AQP4‐IgG or not. We then enrolled 27 NMOSD patients and 17 healthy controls for peripheral blood flow cytometric analyses to validate part of our findings.

**Results:**

In this study, we found multiple possible genetic associations between immune cells and NMOSD. Beyond the well‐recognized roles of T and B cells, diverse myeloid‐lineage cells may also contribute to NMOSD pathophysiology. For AQP4‐IgG seropositive patient, myeloid cells, including dendritic cells (DCs) and the surface molecule CD80, granulocytic myeloid‐derived suppressor cells (MDSCs), and the molecule CX3CR1 may have a protective role in this group. In AQP4‐IgG seronegative patients, molecules like herpesvirus entry mediator (HVEM) exert pathogenic roles in NMOSD. Further flow‐cytometric analysis revealed that the proportion of MDSCs in NMOSD patients was lower, consistent with the MR analysis. However, the phenotypic expression of CX3CR1 in our NMOSD cohort yielded results opposite to those of the MR analysis. Additionally, many immune traits were correlated with the clinical phenotypes of NMOSD patients.

**Conclusio:**

Adaptive immune cells play significant role in NMOSD patients.Certain innate immune cells (e.g., DCs and MDSCs) and surface molecules (e.g., CX3CR1 and CD80) may correlate with certain clinical phenotypes.

## Graphical abstract

This study used two‐sample Mendelian randomization to assess causal relationship of immune cells with NMOSD. We then enrolled 27 NMOSD patients and 17 healthy controls for peripheral blood flow cytometric analyses to validate part of MR findings.

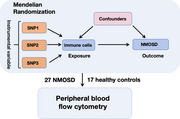

## 1 Introduction

Neuromyelitis optica spectrum disorders (NMOSD) are a rare central nervous system (CNS) demyelinating disease mainly mediated by humoral immune responses (Jarius and Wildemann 2019). Optic neuritis (ON), longitudinally extensive myelitis, and area postrema syndrome (APS) are core clinical manifestations in this disorder (Wingerchuk et al. 2015). The prevalence of NMOSD varies by ethnicity and region, with a much larger proportion in Asians (20%–48%). The characteristics of recurrence and disability have brought a huge disease burden.

Almost 80% of the patients fulfilling the current diagnostic criteria for NMOSD are positive for serum immunoglobulin G (IgG) autoantibodies against aquaporin‐4 (AQP4), which is a water channel protein mainly expressed in the end‐foot processes of astrocytes in the CNS (Jarius and Wildemann 2013; Nielsen et al. 1997). The pathogenic binding of AQP4‐IgG to AQP4 contributes to the formation of typical NMOSD lesions, characterized by astrocytic injury and glial fibrillary acidic protein (GFAP) immunoreactivity, immune cell infiltration, IgG, and activated complement deposition around blood vessels, also triggering secondary damage to oligodendrocytes, axonal dysfunction, and neuronal death (Da Silva et al. 2019).

However, this immunobiological mechanism cannot explain the remaining 20% of AQP4‐IgG negative cases. Studies have shown distinctions in indicators such as GFAP, IL‐6, and other immune agents between serum‐positive and serum‐negative NMOSD patients, suggesting different pathophysiological mechanisms between the two, thereby adding complexity to the immunopathogenesis of the disease (Hyun et al. 2021; “Correction: CSF Cytokine Profile in MOG‐IgG^+^ Neurological Disease is Similar to AQP4‐IgG^+^ NMOSD But Distinct From MS: A Cross‐Sectional Study and Potential Therapeutic Implications” 2019). Recently, serum myelin oligodendrocyte glycoprotein IgG (MOG‐IgG) has been found in some AQP4‐IgG‐negative cases, which has been termed MOG antibody‐associated disease (MOGAD) (Hamid et al. 2017). Therefore, antibody‐negative disease refers to those negative for both AQP4‐IgG and MOG‐IgG (double‐negative NMOSD) in the current consensus (Tieck et al. 2024). However, due to previous issues, seronegative NMOSD refers to AQP4‐IgG‐negative status in this study.

Mendelian randomization (MR) exploits genetic variants influencing the exposure of interest as unbiased proxies for the exposure and minimizes the effects of confounding and reverse causation (Collongues et al. 2019). A MR study has identified potential druggable targets (e.g., COL4A1 and NEU1) for NMOSD by integrating *cis*‐eQTL and large‐scale genome‐wide association study (GWAS) data (Smith and Ebrahim 2003). A more recent study integrated plasma and cerebrospinal fluid protein quantitative trait locus (pQTL) data with GWAS data from NMOSD patients and further identified that plasma CLEC11A and SERPINA1, as well as CSF FAM3B, are associated with NMOSD (Cao et al. 2025). These findings provide a genetic basis for exploring the pathophysiological mechanisms of NMOSD. In this study, we implemented a two‐sample MR analysis using published GWAS data on NMOSD patients from Karol et al. (Meng et al. 2025) and a high‐resolution immune cell profiling GWAS to explore the causal relationship between circulating immune cells and NMOSD (Estrada et al. 2018). We concurrently used peripheral‐blood flow cytometry to validate select immunophenotypes of interest in an NMOSD cohort.

## 2 Methods

### 2.1 Study Design

We explored the causal relationship between 731 immune cell signatures and NMOSD by two‐sample MR analysis. MR uses genetic variations as instrumental variables (IVs), which must meet the following three key assumptions: (1) Genetic variations are directly related to exposure; (2) genetic variations are not associated with potential confounding factors between exposure and outcomes; and (3) genetic variations do not affect pathways beyond the exposure. The statistical data were summarized from individuals of European ancestry in both exposure and outcome datasets to limit the potential bias caused by population stratification.

### 2.2 Exposure Data Sources (GWAS Data Sources for Immune Cells) and Outcome Data Sources (GWAS Data Sources for NMOSD)

Effect estimates for each of the 731 immunophenotypes are publicly available from Orrù et al. (2020), which were conducted on 3757 European individuals by flow cytometry, and approximately 22 million genetic variants were genotyped with high‐density arrays and imputed. Specifically, the immune traits include absolute cell (AC) counts (*n* = 118), median fluorescence intensities (MFI) reflecting surface marker levels (*n* = 389), morphological parameters (MP) (*n* = 32), and relative cell (RC) counts (*n* = 192). The MFI, AC, and RC features include B cells, conventional dendritic cells (cDCs), mature stages of T cells, monocytes, myeloid cells, TBNK (T cells, B cells, and natural killer [NK] cells), and regulatory T cells (Treg) panels, whereas the MP feature includes cDCs and TBNK panels.

The primary outcome for this study was NMOSD, defined by international consensus diagnostic criteria for NMOSD of 2015 (Wingerchuk et al. 2015). Effect estimates of single‐nucleotide polymorphisms (SNPs) associated with NMOSD were obtained from the study of K. Estrada et al. (2018), which involved 215 NMOSD cases and 1244 controls of European ancestry. To investigate whether there are genetic differences among patients with distinct antibody phenotypes, the study also stratified NMOSD into categories including 132 AQP4‐IgG‐seropositive NMOSD (AQP4‐IgG^+^), 83 AQP4‐IgG‐seronegative NMOSD (AQP4‐IgG^−^), and a total of 215 NMOSD cases irrespective of antibody status. In our analysis, we also utilized this stratification to explore the expression of immune traits in NMOSD patients with or without serum AQP4‐IgG.

### 2.3 IVs Selection

For each immune trait derived from the GWAS datasets, SNPs were selected at a *p* value cutoff of 1 × 10^−5^. To ensure that the variants used as IVs were independent, we clumped the SNPs (linkage disequilibrium [LD] *r* ^2^ < 0.001 within a 1000 kb window). The proportion of phenotypic variation explained (PVE) and the *F* statistic were also calculated for each IV to evaluate IV strength and avoid weak instrument bias (Lawlor et al. 2008). To reduce the bias resulting from weak IVs, only SNPs with *F* > 10 were retained for subsequent MR analyses. The power estimation for IVs was set with a type 1 error (*α*) of 0.05 and odds ratios (ORs) derived from the inverse variance weighted (IVW) method.

### 2.4 Patient Information and Sample Collection

#### 2.4.1 Participants

In this study, 29 patients suspected of NMOSD who visited our hospital between July 2024 and April 2025 were enrolled. Finally, 27 patients met the diagnostic criteria outlined in the 2021 Chinese Guidelines for the Diagnosis and Treatment of Neuromyelitis Optica Spectrum Disorders (“Chinese Guidelines for Diagnosis and Treatment of Neuromyelitis Optical Spectrum Disorders (2021 Edition)” 2021). At the same time, we recruited 17 healthy controls (HCs) matched for gender and age. To avoid treatment‐induced bias, patients enrolled in the study had not received B‐cell depletion therapies, including antihuman CD20 monoclonal antibody (mAb) and antihuman CD19 mAb, complement inhibitors, and IL‐6 receptor antagonists, in the past 6 months. Patients who had received intravenous immunoglobulin (IVIG) therapy or plasma exchange within the last month were also excluded. The terms of the Helsinki Declaration for use of patient material were followed throughout the study. For all NMOSD patients, sex, age, disease duration, expanded disability status scale (EDSS) scores, attack status, whether it was the first attack, number of attacks, serum AQP4‐IgG titer, clinical syndromes including ON, myelitis, APS, acute brainstem syndrome (ABS), symptomatic narcolepsy, or acute diencephalic clinical syndrome as well as symptomatic cerebral syndrome, and treatments were included. Because patients with NMOSD often have other autoimmune diseases at the same time, we also recorded the comorbidities of these patients.

#### 2.4.2 Sample Collection and Flow Cytometry

Given the substantial research on T and B cells in NMOSD patients, this study, in conjunction with MR results, focuses on several intriguing molecules, including CX3CR1 and herpesvirus entry mediator (HVEM), as well as innate immune response cells such as DCs and myeloid‐derived suppressor cells (MDSCs). Peripheral blood samples were collected from participants and divided into three equal portions after being mixed evenly for T cells, DCs, and MDSCs (Yang et al. 2018). The antibodies were used according to the manufacturer's instructions and the experimental protocol, including CD3‐FITC (100302), CD4‐PE (100404), CD8‐antigen‐presenting cells (APC)‐Cy7 (100818), CD45‐QB500 (A6015V32), HVEM‐PE‐Cy7 (318809), CX3CR1‐APC (341610), lineage (CD3/CD14/CD19/CD20/CD56)‐FITC (A3432), CD123‐PE (112304), HLA‐DR PerCP‐Cy5.5 (170118), CD80‐PE‐Cy7 (A8936), CD11c‐APC (190314), lineage (CD3/CD19/CD56)‐FITC (A3431), CD33‐PE (A6533), CD15‐PE Cy7 (101512), CD14‐APC (A3175), CD16‐APC‐Cy7 (A6337), and CD11b‐QB450 (A3471). Then, 100 µL of peripheral blood was added to the flow cytometry tube, vortexed thoroughly, and incubated at room temperature in the dark for 15 min. After staining with surface Abs, 1 mL of 1 × lysing solution (BD, 555899) was added to the sample, vortexed to mix, and incubated at room temperature in the dark for another 10 min. Subsequently, centrifuge at 500 × *g* for 5 min, discard the supernatant, then add 1–2 mL of 1 × PBS, and vortex to resuspend the cells. After that, centrifuge again at 500 *g* for 5 min, discard the supernatant, and resuspend in 300–500 µL of PBS for subsequent processing. Stained cells were detected using FACSCanto II 3L8C (BD), and data analysis was conducted with Kaluza version 3.1.

#### 2.4.3 AQP4‐IgG Titer

All NMOSD patients underwent serum AQP4‐IgG titer testing, and the detection of AQP4‐IgG was performed via a cell‐based assay (CBA). Furthermore, NMOSD patients were stratified into four groups based on AQP4‐IgG titer: seronegative, 1–10‐fold, 10–80‐fold, and >80‐fold groups.

It should be noted that the MR analysis was based on GWAS summary data from European ancestry populations, whereas our flow cytometry validation was performed in a Chinese NMOSD cohort. The validation was primarily designed to explore consistency between molecular immune phenotypes and MR‐derived findings, rather than to directly replicate genetic associations.

#### 2.4.4 Statistical Analysis

R 3.5.3 version was used to perform the MR analysis. To evaluate the causal effects of 731 immunophenotypes on NMOSD with different AQP4‐IgG statuses, the IVW method, as well as weighted median‐based and mode‐based methods, was mainly performed by using the “TwoSampleMR” package. The Wald ratio method was used when the number of IVs was less than two, with ORs described as per standard deviation (SD) increase in the levels of risk factor (Burgess et al. 2017; Bowden et al. 2016; Hartwig et al. 2017). If the outcome in the IVW method was significant and no contradictory results were found in the sensitivity analysis, the causal impact of exposure on NMOSD was considered indicative. Heterogeneity of IVs was assessed by Cochran's *Q* statistic, and potential directional pleiotropy was evaluated by MR‐Egger intercept test and MR‐PRESSO global test (Burgess and Thompson 2017). In addition, we also used simple mode, weighted median, and weighted mode to exclude possible horizontal pleiotropic outliers that could substantially affect the estimation results (Verbanck et al. 2018). The leave‐one‐out method was used to assess the effects of individual SNP drivers on the random estimates. Further, the Steiger directionality test was used to determine the direction of causality between exposure and outcome. Finally, funnel and forest plots were performed to assess the symmetry and effect estimates.

SPSS 25.0 was used to carry out the statistical analyses. Categorical data are presented as frequencies and percentages, whereas quantitative data are presented as either medians (interquartile ranges) (IQRs) or means ± SDs if the data follow a normal distribution. To reduce the likelihood of type 1 errors during multiple hypothesis testing, we applied a false discovery rate (FDR) technique to adjust the IVW results after per‐analysis of the 731 immune cells with a single outcome. A pFDR value of less than 0.05 indicates a significant causal relationship. Student's *t*‐test (for normally distributed data) or Mann–Whitney *U* test (for non‐normally distributed data) was used to compare differences. The associations between variables were assessed via Spearman's correlation tests and linear regression models. To account for potential confounding factors, multivariable linear regression models were further constructed to assess the independent associations between immune cell subsets and disease status, adjusted for age, sex, treatment history, time since treatment discontinuation, and disease activity status. A two‐tailed *p* value of less than 0.05 was deemed statistically significant.

## 3 Results

### 3.1 Demographic and Clinical Characteristics of Participants

There were 24 females (88.89%) in the NMOSD cohort, with a median age of 41.00 (31.50–65.75) years. In the NMOSD group, only three patients were experiencing their first onset, and eight cases were in the attack phase at the time of enrollment. During blood collection, seven patients were admitted without any medication, and no patients received glucocorticoids or immunosuppressive treatments. Additionally, 20 patients received biologic therapy, including 7 who were treated with anti‐CD20 mAb rituximab, 10 who were treated with anti‐CD19 mAb inebilizumab, 2 who were treated with eculizumab regularly, and 1 who was treated with other agents. The last treatment for all these patients was at least 6 months before enrollment. In fact, all these patients had one or more episodes with serum AQP4‐IgG positivity during the disease. However, in line with the MR analysis approach of this study, we examined the serum AQP4‐IgG status of these patients at the time of enrollment and found that four patients were negative for serum AQP4‐IgG. Seven NMOSD cases had comorbid autoimmune diseases, including Sjogren's syndrome, Hashimoto's thyroiditis, and nephrotic syndrome. Interestingly, two patients had other autoimmune diseases of the nervous system. One patient had myasthenia gravis (MG) positive for acetylcholine receptor (AChR) antibody, whereas the other had stiff‐person syndrome (SPS) and was positive for glutamate decarboxylase 65 (GAD65) IgG in the CSF, as seen in Table 1 and Table S9.

**TABLE 1:** Demographic and clinical characteristics of participants.

	NMOSD (*n* = 27)	HC (*n* = 17)
Gender (females, %)	24.00 (88.89%)	15.00 (88.24%)
Age (years, median [IQR])	41.00 (31.50–65.75)	35.00 (28.50–51.50)
Attack status (*n*, %)	8 (29.63%)	NA
Number of relapses	2.50 (1.00–4.00)	NA
First episode (*n*, %)	3 (11.11%)	NA
EDSS score	2.50 (2.00–4.00)	NA
Course of disease (years)	5.00 (1.46–9.00)	NA
Treatment (*n*, %)	20 (74.07%)	NA
Inebilizumab	10 (50.00%)	NA
Anti‐CD20 mAb	7 (35.00%)	NA
Eculizumab Others	2 (10.00%) 1 (5.00%)	NA NA
Serum AQP4‐IgG (positivity, %)	23 (85.19%)	NA
Comorbid ADs (*n*, %)	7 (25.93%)	NA

Abbreviations: ADs, autoimmune diseases; AQP4‐IgG, aquaporin‐4 immunoglobulin G; EDSS, expanded disability status scale; HC, healthy controls; IQRs, interquartile ranges; mAb, monoclonal antibody; NMOSD, neuromyelitis optica spectrum disorders.

### 3.2 The Causal Effect of Immunophenotypes on NMOSD

This analysis revealed no heterogeneity among the data, as Cochran's *Q* test was not statistically significant (*p* > 0.05). Both the MR‐PRESSO global test and the MR‐Egger intercept test yielded *p* values above the 0.05 threshold, indicating no significant horizontal pleiotropy bias. Leave‐one‐out plots showed that the MR results were not affected by any single SNP (Tables S5 and S6).

First, we analyzed the immune cell phenotypes that exhibited statistically significant causal relationships with the outcome across the three groups (pFDR < 0.05). We found that both the total 215 NMOSD patients (OR = 0.4, 95% CI: 0.25–0.64, *p* = 0.02, pFDR = 0.03; IVW) and AQP4‐IgG‐seropositive NMOSD (OR = 0.29, 95% CI: 0.16–0.53, *p* = 0.02, pFDR = 0.01; IVW) were genetically associated with effector memory CD4^−^CD8^−^ T cell absolute count. However, Steiger's directionality test failed to confirm the hypothesized causal direction (exposure → outcome) (Table S8).

In addition, based on the *p* value results, we have separately presented the top‐ranked results of some immune cells of interest across the three groups.

### 3.3 The Exploratory Causal Effect of Immunophenotypes on Total 215 NMOSD

**FIGURE 1:**
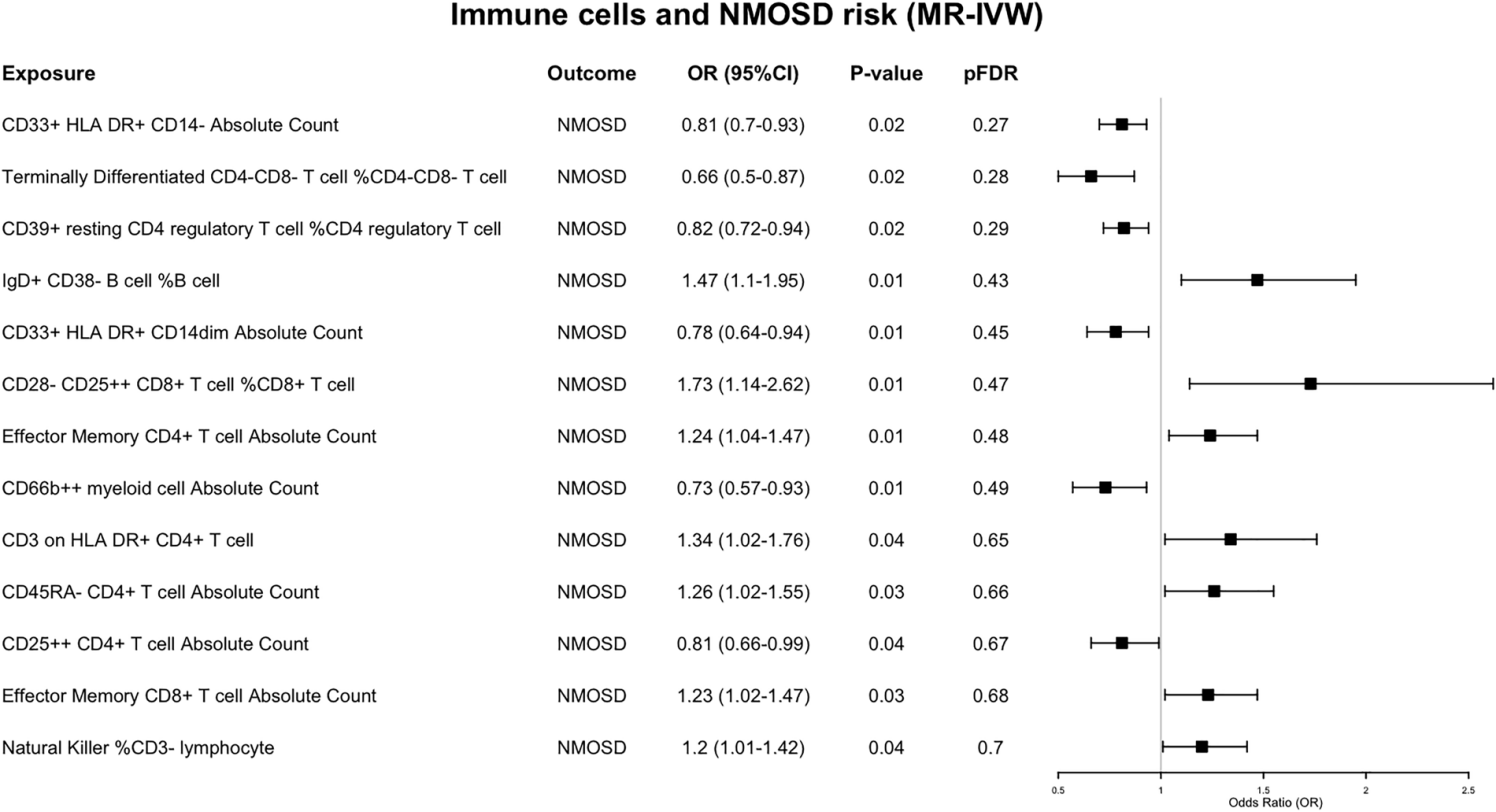
Mendelian randomization estimates of the association between 731 immune traits and risk of 215 NMOSD patients. CI, confidence interval; FDR, false discovery rate; IVW, inverse variance weighted; MR, Mendelian randomization; NMOSD, neuromyelitis optica spectrum disorders; OR, odds ratio.

The exploratory causal estimates of immunophenotypes on NMOSD patients, regardless of AQP4 antibody status. We found that several immune indicators may be potential risk factors for NMOSD though no statistically significant differences were observed after multiple correction by FDR, as shown in Figure 1. As to other immune traits with *p* < 0.01, including IgD^+^CD38^−^ B cell %B cell (OR = 1.47, 95% CI: 1.10–1.95, *p* = 0.01; IVW), CD33^+^HLA DR^+^CD14dim absolute count (OR = 0.78, 95% CI: 0.64–0.94, *p* = 0.01; IVW), effector memory CD4^+^ T cell absolute count (OR = 1.24, 95% CI: 1.04–1.07, *p* = 0.01; IVW), and CD66b^+^^+^ myeloid cell absolute count (OR = 0.4, 95% CI: 0.25–0.64, *p* = 0.02; IVW), as shown in Figure 1 and Table S1.

### 3.4 The Exploratory Causal Effect of Immunophenotypes on AQP4‐IgG‐Seropositive NMOSD

**FIGURE 2:**
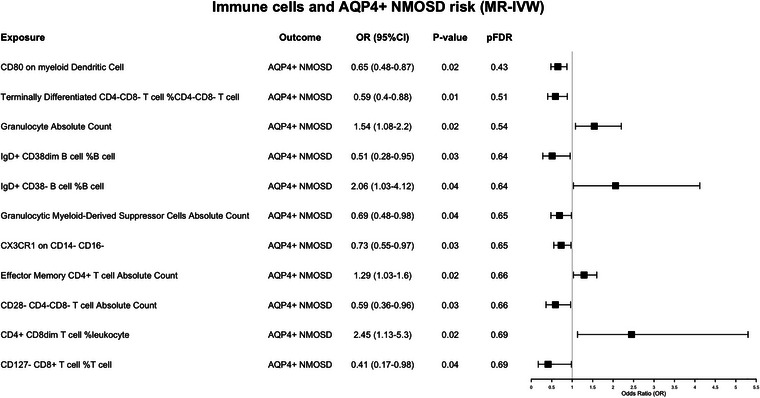
Mendelian randomization estimates of the association between 731 immune traits and risk of 132 AQP4‐IgG‐seropositive NMOSD. AQP4, aquaporin‐4; CI, confidence interval; FDR, false discovery rate; IVW, inverse variance weighted; MR, Mendelian randomization; NMOSD, neuromyelitis optica spectrum disorders; OR, odds ratio.

For patients with AQP4‐IgG, we found that the following immune molecules may be genetically involved in the pathophysiological process of AQP4‐IgG^+^ NMOSD. The cells with *p* value ≤0.01 are terminally differentiated CD4^−^CD8^−^ T cell %CD4^−^CD8^−^ T cell (OR = 0.59, 95% CI: 0.40–0.88, *p* = 0.01; IVW). CD80 on myeloid DCs (OR = 0.65, 95% CI: 0.48–0.87, *p* = 0.02; IVW), CX3CR1 on CD14^−^CD16^−^ (OR = 0.73, 95% CI: 0.55–0.97, *p* = 0.03; IVW), and granulocytic MDSCs absolute count (OR = 0.69, 95% CI: 0.48–0.98, *p* = 0.04; IVW) may suggest a beneficial role for NMOSD patients. All results are shown in Figure 2 and Table S1.

### 3.5 The Exploratory Causal Effect of Immunophenotypes on AQP4‐IgG‐Seronegative NMOSD

**FIGURE 3:**
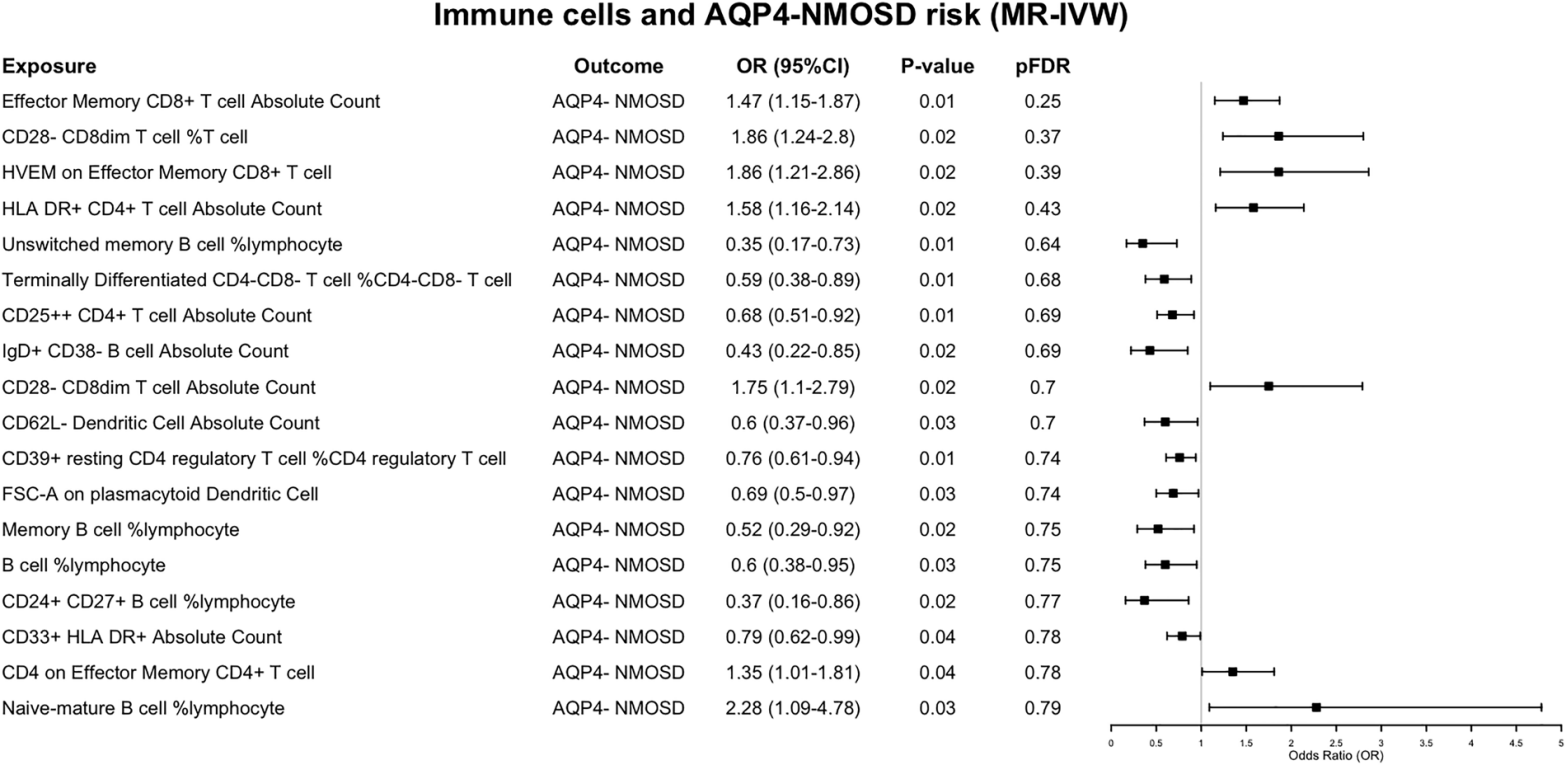
Mendelian randomization estimates of the association between 731 immune traits and risk of 83 AQP4‐IgG‐seronegative NMOSD. AQP4, aquaporin‐4; CI, confidence interval; FDR, false discovery rate; IVW, inverse variance weighted; MR, Mendelian randomization; NMOSD, neuromyelitis optica spectrum disorders; OR, odds ratio.

Accordingly, NMOSD with negative antibodies has a distinct clinical and epidemiological profile, a unique pathogenesis, and different immunological phenotypes, as found in our study. The risk factors for antibody‐negative NMOSD were far from uniform compared to those for AQP4‐IgG^+^ NMOSD. In this cohort, the possible risk factors are as follows: effector memory CD8^+^ T cell absolute count (OR = 1.47, 95% CI: 1.15–1.87, *p* = 0.01; IVW), CD28^−^ CD8dim T cell %T cell (OR = 1.86, 95% CI: 1.24–2.8, *p* = 0.02; IVW), HLA‐DR^+^CD4^+^ T cell absolute count (OR = 1.58, 95% CI: 1.16–2.14, *p* = 0.02; IVW), and naive‐mature B cell %lymphocyte (OR = 2.28, 95% CI: 1.09–4.78, *p* = 0.03; IVW), and so on. The immune traits, including CD39^+^ resting CD4 regulatory T cell % (OR = 0.76, 95% CI: 0.61–0.94, *p* = 0.01; IVW), unswitched memory B cell %lymphocyte (OR = 0.35, 95% CI: 0.17–0.73, *p* = 0.01; IVW), CD62L^−^ DC absolute count (OR = 0.60, 95% CI: 0.37–0.96, *p* = 0.03; IVW), and FSC‐A on plasmacytoid DCs (pDCs) (OR = 0.69, 95% CI: 0.50–0.97, *p* = 0.03; IVW), suggest a trend toward a protective effect in this subgroup though with insufficient evidence, as shown in Figure 3 and Table S1. There were still no differences after FDR correction.

Furthermore, the causal direction between immune traits and disease was confirmed by the Steiger test (Table S8). Detailed MR results, sensitivity analyses, and leave‐one‐out plots are provided in Tables S1–S6. No heterogeneity (Cochran's *Q* test, *p* > 0.05) or significant horizontal pleiotropy (MR‐PRESSO global test and MR‐Egger intercept test, both *p* > 0.05) was detected, and leave‐one‐out analyses confirmed that no single SNP drove the results.

### 3.6 The Association and Difference of Immunophenotypes Among Three Groups

Overall, the potential risk factors for all 215 NMOSD patients were a collection of immunological phenotypes from both antibody‐positive and antibody‐negative cases, as shown in Figure 1 and Table S1. The similarities among immune cells with potential relationships between patients with different antibody states are concentrated in effector memory CD4^+^ T cells, effector memory CD8^+^ T cells, terminally differentiated CD4^−^CD8^−^ T cells, %CD4^−^CD8^−^ T cell, as well as B cells at different differentiation stages. However, there are still significant differences in immunological characteristics between the two groups.

In terms of T cell phenotypes, AQP4^+^ NMOSD was linked to CD4‐biased and double‐negative T cell subsets like effector memory CD4^+^ T cells and CD28^−^CD4^−^CD8^−^ T cells, whereas AQP4^−^ NMOSD showed possible associations with a broader range of effector, activated, and regulatory CD8/CD4 T cell subsets, including effector memory CD8^+^ T cells, HLA‐DR^+^ CD4^+^ T cells, CD25^++^ CD4^+^ T cells, and CD39^+^ resting Tregs. For B cell phenotypes, AQP4^+^ NMOSD was specifically associated with IgD^+^ CD38dim and IgD^+^ CD38^−^ B cell subsets, suggesting a role for immature/naive B cell homeostasis. In comparison, AQP4^−^ NMOSD showed extensive B cell‐related associations, including unswitched memory B cells, total B cells, memory B cells, CD24^+^CD27^+^ B cells, and naive‐mature B cells, indicating widespread dysregulation across naive, memory, and mature B cell compartments (Figures 2 and 3).

Innate immune associations differed markedly between AQP4^+^ and AQP4^−^ NMOSD with little overlap. AQP4^+^ NMOSD was linked to myeloid/granulocytic lineages, including myeloid DCs (CD80^+^), granulocytes, granulocytic MDSCs, and CX3CR1^+^ CD14^−^CD16^−^ myeloid cells. In contrast, AQP4^−^ NMOSD was associated with pDCs (FSC‐A) and CD33^+^HLA‐DR^+^ myeloid cells, indicating myeloid‐driven inflammation in AQP4^+^ NMOSD and pDC/monocyte/myeloid abnormalities in AQP4^−^ NMOSD (Figures 2 and 3).

### 3.7 Abnormality of Immunophenotypes in NMOSD

**FIGURE 4:**
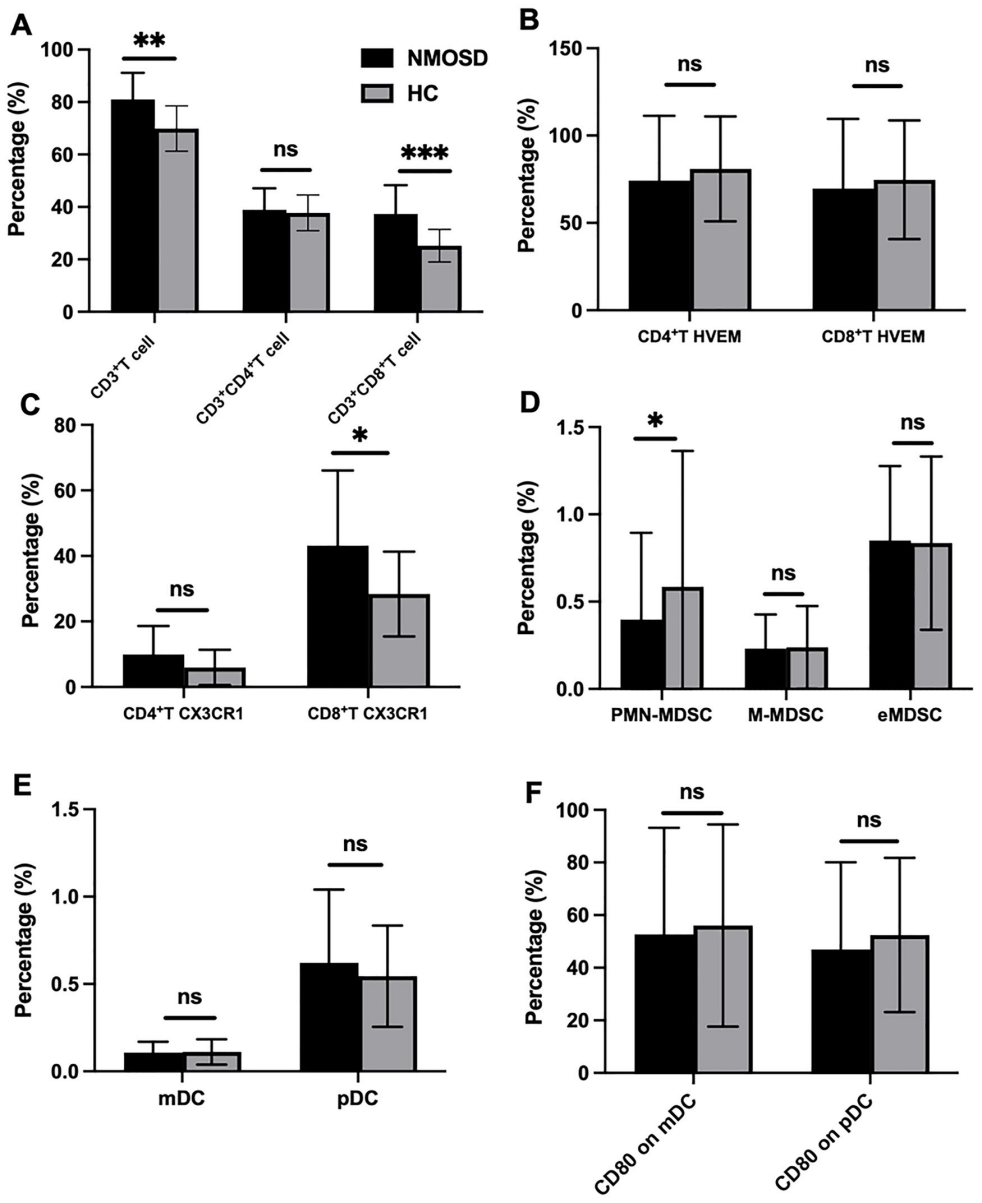
Abnormality of partial immunophenotypes in NMOSD. (A) The percentage of CD3^+^T cell, CD3^+^CD4^+^T cell, and CD3^+^CD8^+^ T cell between NMOSD and HC, (B) the percentage of HVEM on CD4^+^T cell and HVEM on CD8^+^T cell between NMOSD and HC, (C) the percentage of CX3CR1 on CD4^+^T cell and HVEM on CD8^+^T cell between NMOSD and HC, (D) the percentage of MDSC between NMOSD and HC, (E) the percentage of dendritic cells between NMOSD and HC, and (F) the percentage of CD80 on dendritic cells between NMOSD and HC. eMDSC, early‐stage myeloid‐derived suppressor cells; HC, healthy controls; HVEM, herpesvirus entry mediator; mDC, myeloid‐derived dendritic cells; M‐MDSC, monocytic myeloid‐derived suppressor cells; NMOSD, neuromyelitis optica spectrum disorders; ns, not significant; pDC, plasmacytoid dendritic cells; PMN‐MDSC, polymorphonuclear myeloid‐derived suppressor cells. *p < 0.05; **p < 0.01.

As discussed above, the flow cytometric gating strategy was used for analysis of CD3^+^CD4^+^ T cells, CD3^+^CD8^+^ T cells, CX3CR1 on T cells, HVEM on T cells, polymorphonuclear (PMN‐MDSCs), monocytic MDSCs (M‐MDSC), early‐stage MDSC (eMDSC), pDCs, and myeloid dendritic cell (mDCs) as well as CD80^+^ DCs. First, the percentages of CD3^+^ T cells and CD3^+^CD8^+^ T cells were observed to be significantly elevated in NMOSD patients (26.74% vs. 15.76%, *p* < 0.01 and 28.30% vs. 13.29%, *p* < 0.001, respectively; Figure 4A). After adjustment for sex, age, disease status, and treatment via multivariable linear regression, the differences remained statistically significant (*p* = 0.043 and *p* = 0.033). The percentages of CX3CR1 on CD4^+^ T cells and CD8^+^ T cells were both higher in the NMOSD group, and the difference in CX3CR1 on CD8^+^ T cells was statistically significant by nonparametric testing (25.78% vs. 17.29%, *p* < 0.05, Figure 4C). However, neither difference reached statistical significance after adjustment by multivariable linear regression. Patients with NMOSD exhibited downregulation of PMN‐MDSCs (0.41% vs. 0.59%, *p* < 0.05), though there were no significant differences for M‐MDSCs and eMDSCs (Figure 4D). Moreover, none of these three differences reached statistical significance after adjustment by multivariable linear regression. Finally, the populations of HVEM on T cells, mDCs, pDCs, and CD80 on DCs did not differ significantly between the two groups (Figure 4B,E,F).

### 3.8 Associations Between Immunophenotypes and Clinical Characteristics in NMOSD

To investigate whether the clinical characteristics of NMOSD patients would affect the peripheral blood immune phenotypes mentioned above, we conducted a correlation analysis and stratified analysis. Spearman correlation analysis showed that HVEM on CD4^+^ T cells and CD8^+^ T cells both had a positive correlation with symptomatic cerebral syndrome (*r* = 0.538, *p* < 0.01 and *r* = 0.603, *p* = 0.001, respectively). CX3CR1 on CD8^+^ T cell was positively correlated with EDSS score (*r* = 0.526, *p* < 0.01). Age also significantly affected the levels of CX3CR1 on CD8^+^ T cells in peripheral blood (*r* = 0.752, *p* < 0.001). The levels of eMDSCs were negatively correlated with the occurrence of ON (*r* = −0.535, *p* < 0.01), which may indicate the immunosuppressive effect of these cells. CD80 on DCs was positively correlated with the number of core symptoms for NMOSD, though the correlation was weak. AQP4‐IgG titer was not correlated with these immune traits (Table S10).

**FIGURE 5:**
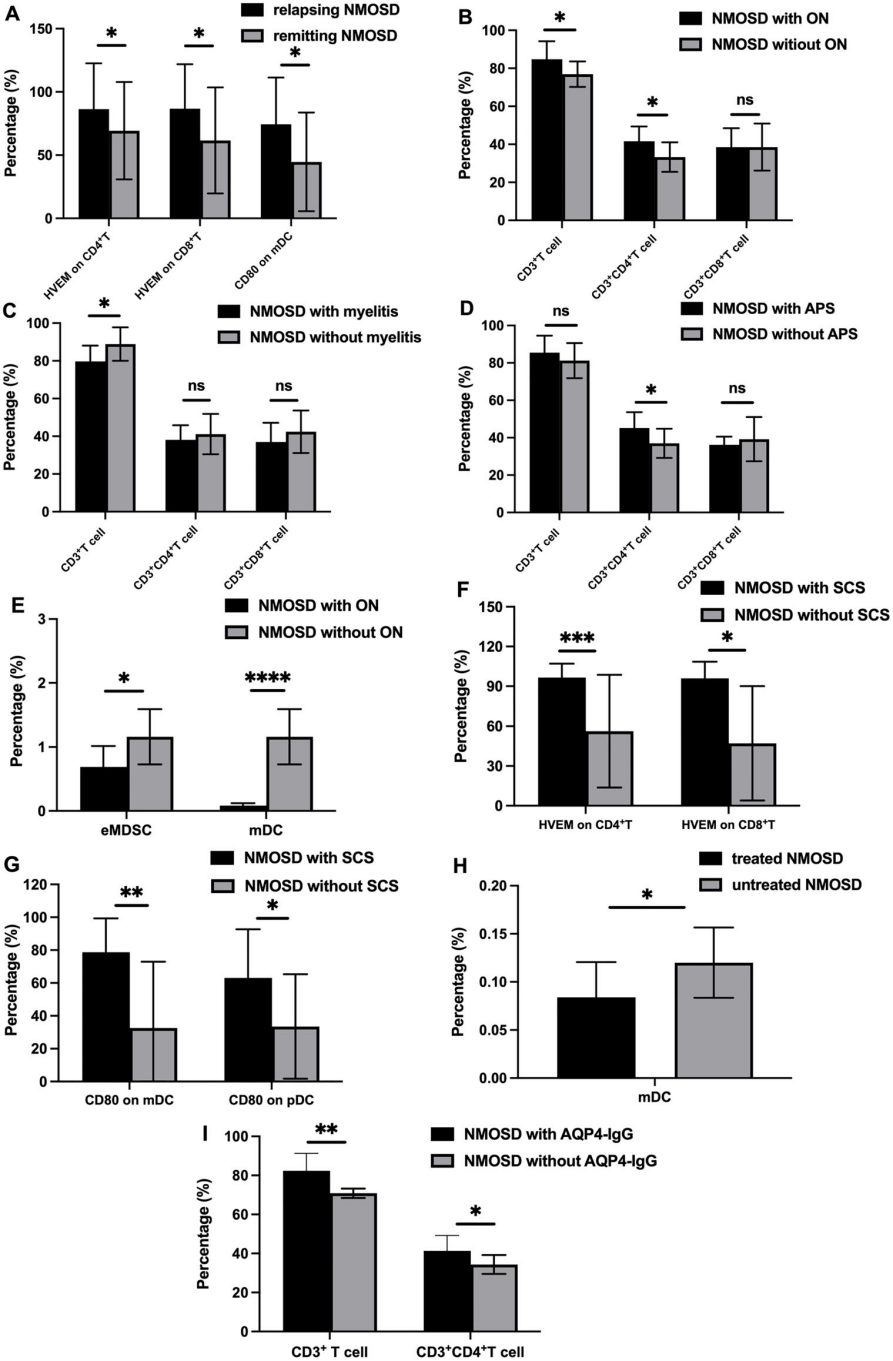
Abnormality of immunophenotypes in NMOSD subgroups. (A) The percentages of HVEM on CD4^+^T cell, HVEM on CD8^+^T cell, and CD80 on myeloid dendritic cells NMOSD were higher in NMOSD patients during the attacking period, (B) differential expression of T cells in NMOSD patients with or without optic neuritis, (C) differential expression of T cells in NMOSD patients with or without myelitis, (D) differential expression of T cells in NMOSD patients with or without area postrema syndrome, (E) the percentage of eMDSC and mDC between NMOSD with or without optic neuritis, (F) the percentage of HVEM on T cells between NMOSD with or without symptomatic cerebral syndrome, (G) the percentage of CD80 on DCs between NMOSD with or without symptomatic cerebral syndrome. (H) the percentage of mDC in treated and untreated NMOSD, and (I) the percentages of CD3^+^T cells and CD3^+^CD4^+^T cells were elevated in NMOSD patients with AQP4‐IgG. APS, area postrema syndrome; AQP4‐IgG, aquaporin 4 immunoglobulin G; eMDSC, early‐stage myeloid‐derived suppressor cells; HVEM, herpesvirus entry mediator; mDC, myeloid dendritic cells; NMOSD, neuromyelitis optica spectrum disorders; ns, not significant; ON, optic neuritis; pDC, plasmacytoid dendritic cells; SCS, symptomatic cerebral syndrome. *p < 0.05; **p < 0.01; ***p < 0.001; ****p < 0.0001.

Further analysis revealed that multiple clinical phenotypes and interventions may have an impact on the immune phenotypes. First, the expression of HVEM molecules on T cells was elevated in patients with active NMOSD. CD80 on mDCs was also elevated in these patients (18.5% vs. 10.86%, *p* < 0.05). NMOSD patients presenting with ON and APS exhibited elevated expression of CD3^+^CD4^+^ T cells (15.29% vs. 8.13%, *p* < 0.05 and 18.17% vs. 11.37%, *p* < 0.05, respectively; Figure 5). Interestingly, NMOSD patients presenting with myelitis exhibited decreased levels of CD3^+^ T cells in peripheral blood. In addition to the HVEM molecule, the expression of CD80 molecules on DCs was also significantly increased in NMOSD patients presenting with cerebral syndrome. NMOSD patients with AQP4‐IgG had higher expression of CD3^+^ T cells and CD3^+^CD4^+^ T cells. Whether the disease was the first episode or not does not appear to impact the immune phenotypes. Additionally, no significant differential expression of these immune phenotypes was observed in patients with other concomitant autoimmune diseases.

Although we attempted to minimize the impact of treatments on the peripheral blood immune cells of NMOSD patients, differences in the expression of certain immune cells still existed in these patients who had received specific treatments. First, the level of mDCs was decreased in treated NMOSD patients in our cohort (Figure 5). We also compared whether different treatment regimens would affect these indicators. In patients treated with inebilizumab, the expression of CD80 on DCs was reduced, but this was not observed in other immune phenotypes.

## 4 Discussion

Since the boundary of NMO was extended to NMOSD in 2015, those without AQP4‐IgG have entered the field of vision and been receiving increasing research attention. Like AQP4‐IgG^+^ NMOSD patients, the inflammatory response in AQP4‐IgG^−^ NMOSD is also increased, as reflected by significantly elevated serum proinflammatory cytokines and complement activation (Hakobyan et al. 2017). However, several studies have found that antibody‐negative patients differ from positive ones in aspects of epidemiology, clinical phenotypes, and radiological and laboratory characteristics, which may indicate a heterogeneous immune background. Regretfully, there are few conclusions regarding the discrepancies between the two subgroups with respect to the pathophysiology. In our study, we attempt to answer this question in terms of immunological cell profiling by two‐sample MR analysis. Although the results, which did not reach statistical significance after FDR correction, were not entirely satisfactory, our study identified some interesting findings.

The adaptive immune response comprises T cell‐mediated cellular immunity and B cell‐mediated humoral immunity, with intricate crosstalk between the two. It is important to note that the pathological process of NMOSD begins with the activation of pathogenic T cells, especially CD4^+^ T cells. These CD4^+^ T cells in the NMOSD lesions are activated via antigen presentation by macrophages and/or DCs. Then, they open the BBB, allowing the large‐scale entry of antibodies and complements, which permits the AQP4‐IgG to bind with astrocytes and leads to complement deposition (Bradl et al. 2009; Zeka et al. 2015). In addition to CD4^+^ T cells, CD8^+^ T cells have also been found to be involved in the immunopathogenesis of NMOSD (Lucchinetti et al. 2002). Studies have reported that the levels of the transcription factor T‐bet in circulating proinflammatory CD8^+^ T cells are significantly elevated in NMOSD and contribute to increased terminal differentiation of CD8^+^ T cells and high expression of cytotoxic granzyme B (Shi et al. 2021, 2022). In the MR analysis, CD4^+^ T cells, especially those in the effector memory state, showed suggestive evidence for a potential causal association to NMOSD in both AQP4‐IgG^+^ and ‐IgG^−^ groups. However, we still found that AQP4‐IgG^+^ NMOSD had a higher level of CD4^+^ T cells. Additionally, we found that CD4^+^ T cells may be involved in clinical phenotypes in NMOSD patients, such as ON and APS. In our cohort, CD8^+^ T cells were significantly elevated in the NMOSD group compared with the control group. Our MR study found that different types of CD8^+^ T cells may be involved in the pathological process of NMOSD, regardless of antibody status. We also found that effector memory CD8^+^ T cells may be a risk factor for AQP4^−^ NMOSD. Shi et al. (2021) found that the proportion of effector memory CD8^+^ T cells (TEM) in the peripheral blood of NMOSD patients was significantly increased, whereas the frequency of naïve CD8^+^ (TN) cells decreased, suggesting a skewed differentiation of CD8^+^ T cells toward the effector memory subset.

Although B cells were not the focus of our validation, the MR analyses still revealed some intriguing findings. We found that B cells played different roles and exhibited varying proportions in AQP4‐IgG^+^ and—IgG^−^ NMOSD groups. In previous studies, the proportions of B cell populations in NMOSD were found to shift from regulatory to a more activated, memory B cell phenotype (Quan et al. 2013). When abnormally activated memory B lymphocytes escape immunologic surveillance, they differentiate into plasmablasts and plasma cells that produce AQP4‐IgG. Undoubtedly, AQP4‐IgG^+^ patients harbor an important humoral response, implicating a clear role for B‐lymphocytes in the disease pathophysiology (Chu et al. 2023). For AQP4‐IgG^−^ NMOSD, our research suggests a different immune profile. We found that memory B cells and unswitched memory B cells may play a protective role in AQP4‐IgG^−^ NMOSD. A 2024 review indicated that elevated double‐negative B cells and plasmablasts, but not memory B cells, represent the characteristic abnormalities in NMOSD (Tieck et al. 2024). Anti‐CD20 therapy, which depletes memory B cells, also shows poor response in some AQP4‐negative patients, suggesting that the pathogenesis of these patients is independent of memory B cell‐driven humoral immunity and instead requires intact memory B cells to maintain immune homeostasis. CD24^+^CD27^+^ B cells represent an important regulatory B cell (Breg) subset, which exerts immunosuppressive functions mainly by secreting anti‐inflammatory cytokines such as IL‐10, and our MR results suggest this subset may contribute to protective mechanisms in AQP4‐IgG^−^ NMOSD.

HVEM, also known as CD272 or TNFRSF14, is a type 1 transmembrane glycoprotein that belongs to the TNFRSF (tumor necrosis factor receptor superfamily) and is expressed on various cells (Ware and Šedý 2011). HVEM can exert complex bidirectional effects, either stimulatory or inhibitory, on cellular activity (Bodmer et al. 2002; Shaikh et al. 2001). HVEM‐LIGHT signaling plays a vital role in regulating T cell activation, expansion, and cytokine production, thereby displaying autoreactive potential and leading to excessive tissue destruction (Tan and Zhou 2005). In the MR analysis, we found that HVEM expressed on effector memory CD8^+^ T cells has been linked with AQP4‐IgG^−^ NMOSD patients. Additionally, a recent study found that TNFRSF9 (CD137) also showed a remarkable positive correlation with AQP4^+^ NMOSD by MR analysis, which also highlights the critical role of the TNFRSF in the pathophysiological processes of NMOSD (Chen et al. 2024). However, we found no significant difference in HVEM expression on T cells between HCs and NMOSD patients, regardless of AQP4‐IgG serostatus. Given the promising roles of HVEM in cancer immunotherapies, we still believe that HVEM merits further in‐depth investigation in NMOSD and other CNS autoimmune diseases. CX3CR1, the receptor for CX3CL1 (FKN), is a chemokine that is predominantly expressed in the CNS and localized on neuronal cells (Liu et al. 2021). Additionally, expression of CX3CR1 is also observed across a diverse range of immune cell types, including T cells, monocytes, NK cells, and DCs. FKN/CX3CR1 exerts neuroprotective and neurotrophic effects on neurons by regulating synaptic function and microglial activation. In our MR analysis, the marker CX3CR1 showed suggestive evidence of a beneficial role in NMOSD patients, especially in the AQP4‐IgG^+^ group. However, we found the levels of CX3CR1^+^CD8^+^ T cells were higher in the NMOSD group by peripheral blood flow cytometry, though no statistically significant difference was observed after adjustment for confounding factors. First, the MR data were obtained from European populations, whereas our study was performed in a small Chinese cohort, and ethnic differences may lead to distinct genetic regulation and immune phenotypes. Second, MR reflects long‐term genetic predisposition, whereas flow cytometry measures dynamic expressions that can be affected by disease activity, treatment, and clinical covariates. Third, the genetic variants used in MR may act as eQTLs in a cell‐type‐ and disease‐specific manner, which may not fully represent the surface expression of CX3CR1 on peripheral CD8^+^ T cells in our patients. Moreover, after adjustment for confounders, the differences in CX3CR1^+^ T cell subsets did not reach statistical significance. Together, these factors account for the inconsistency between MR estimates and phenotypic observations. In fact, CX3CR1^+^CD8^+^ T cells have been widely studied in cancer patients, and these cells have shown unparalleled cytotoxic properties and play important roles in leukocyte migration (Zwijnenburg et al. 2023). Additionally, CX3CR1^+^CD8^+^ T cells can release more granzyme A, granzyme B, and perforin, which may enhance anti‐tumor efficacy but may also result in greater immune damage in some diseases, such as NMOSD (Zwijnenburg et al. 2023). Therefore, the biological function of FKN/CX3CR1 involves a complex regulatory mechanism and is worthy of further study in NMOSD patients.

The innate immune system components, such as tissue‐resident macrophages, DCs, mast cells, circulating phagocytes, and complement molecules, represent the first line of defense against invading pathogens or malignancies. Myeloid cells have multiple immunological functions, including antigen presentation, phagocytosis, and cytokine production. In our study, we were also surprised to find that some immune cells may exert a protective effect in NMOSD, which may serve as targets for further research. DCs are professional APCs, which present antigens to T cells via MHC I/II and co‐stimulatory molecules and therefore represent an intersection of the innate and adaptive immune systems (McDonnell et al. 2010). In recent years, DCs can be divided into three major categories, including cDCs, also known as mDCs in previous studies, pDCs, and monocyte‐derived DCs (mo‐DCs) (Bachem et al. 2010). In most studies, cDCs are the main APCs that activate encephalitogenic T cells, licensing them to cross the BBB and invade the CNS parenchyma (Collin and Bigley 2018). These DCs can activate self‐antigen‐specific naïve T cells in peripheral lymph nodes and subsequently promote their differentiation into effector Th1 and Th17 cells in NMOSD. However, there are still some studies that claim the neuroprotective potential of DCs. It has also been reported that pDCs in the CNS, with an accumulation of Tregs, play an important role in tolerance induction and therefore suppress chronic inflammation. The MR study found that pDCs and mDCs (CD80^+^) may play protective roles in AQP4‐IgG^−^ and AQP4‐IgG^+^ NMOSD, respectively, despite not passing stricter statistical correction. However, flow cytometry analysis did not reveal differences in the expression of different DC subtypes between NMOSD and HCs, as well as among NMOSD patients with different antibody profiles. Additionally, we found that different DCs were related to NMOSD clinical phenotypes. The levels of mDCs were significantly elevated in NMOSD without ON, revealing the distinct pathogenesis and therapeutic targets of these patients. In addition to the corresponding DC phenotype transition that can produce neuroprotective effects, we also found that surface markers of DCs can produce similar effects. CD80, as a surface molecule of DCs, has been reported to alleviate allergic diseases, IBD, and Crohn's disease (Mundt et al. 2019; Jostins et al. 2012). It has been reported that CD80^+^ DCs produce high levels of IL‐10 and indoleamine 2,3‐dioxygenase, which induces an increase in the production of tolerogenic regulatory type 1 cells and inhibits inflammatory responses (Ferreira et al. 2017). Furthermore, CD80^+^ DC‐derived exosomes have the capacity to inhibit CD8^+^ T cell activation, proliferation, and adhesion in vitro (Koorella et al. 2014). Similarly, there was no significant difference in the expression of CD80^+^ DCs between NMOSD patients and controls in our cohort. We found that this molecule may be associated with clinical phenotypes such as cerebral syndrome in NMOSD patients; however, the detailed mechanisms of DCs in NMOSD remain incompletely understood, even though DCs play an important role in the immune response.

MDSCs are a large group of bone marrow‐derived cells with potential immunosuppressive activity, and their role in tumor immunity is increasingly being elucidated. In addition, they have also been studied in diseases such as chronic inflammation, autoimmune diseases, and graft‐versus‐host diseases. MDSCs consist of three groups of cells based on their morphological and phenotypic features, termed granulocytic or PMN‐MDSCs, M‐MDSCs, and eMDSC. Although MDSCs can regulate various immune cells, their main function is to inhibit T cell proliferation (Cui et al. 2022). MDSCs can also promote regulatory T cell proliferation and inhibit various CD8^+^ T cell expansion pathways. By releasing various soluble regulatory factors such as IL‐10 and TGF‐β, engaging in metabolic crosstalk as well as through cell‐to‐cell contact, MDSCs promote the induction, proliferation, and activation of Tregs (Melero‐Jerez et al. 2016). Aside from T cells, studies have also found that CNS PMN‐MDSCs can control the aggregation and activation of B cells (Haist et al. 2021). In MS patients, the frequencies of MDSCs in relapsing patients were significantly higher than those in stable MS patients (Knier et al. 2018). It was also shown that there was a significant accumulation of MDSCs in EAE models, in which mMDSCs promote remyelination in EAE by enhancing oligodendrocyte precursor cell (OPC) survival, proliferation, and differentiation (Iacobaeus et al. 2018). We found that the level of PMN‐MDSCs was decreased in NMOSD patients, which was in accordance with the MR analysis. We also found that these cells were decreased in NMOSD presenting with optic neuritis. To date, no studies have investigated the relationship between MDSCs and NMOSD. Combining previous research results as well as the powerful immunosuppressive effects of MDSCs in various immune backgrounds, our results may open the door for the future use of MDSCs as biomarkers for NMOSD (Melero‐Jerez et al. 2021).

However, we must bear in mind that AQP4‐IgG may become undetectable at different stages in NMOSD, so the AQP4‐IgG^−^ group in our study should be interpreted with this caveat. Moreover, with growing understanding of MOGAD, some AQP4‐IgG^−^ patients may have MOGAD but remain unrecognized owing to technical limitations. These two entities are currently regarded as distinct disease categories. Finally, we utilized MR to attempt to identify genetic associations of relevant immune cells in NMOSD patients. However, due to the complexity of actual situations, including the impact of treatment on immune traits and responses, there is still a long way to go before the relevant conclusions can be applied.

It still needs to be acknowledged that there are certain limitations in the MR analysis in this study. Although we assessed the heterogeneity and validity among IVs using a variety of methods, some IVs may be subject to bias, which could affect the accuracy of the causal estimation. Additionally, although we conducted sensitivity analyses using multiple methods, the results from different methods may vary, which may indicate the presence of some unidentified biases. Moreover, potential population stratification should be acknowledged. The present MR analysis was based on GWAS data from European populations, whereas our in‐house flow cytometry validation cohort included Chinese patients. Differences in genetic architecture, allele frequencies, environmental exposures, and clinical profiles between the two ancestries may limit the direct interpretability of the cross‐population validation. Nevertheless, the flow cytometry analysis was performed to assess whether immune cell phenotypic patterns were consistent with our MR results, rather than to validate genetic effects per se, thus providing preliminary translational evidence in a non‐European (Chinese) population. Therefore, future studies with ancestry‐matched genetic and phenotypic data are warranted. Lastly, the relatively small sample size of the flow cytometry cohort may limit the statistical power to detect significant differences. Additionally, previous exposure to immunomodulatory or biologic agents, even after a 6‐month washout period, could potentially influence immune cell profiles. Post hoc power analysis was conducted using G*Power 3.1 for two independent sample *t*‐tests. Assuming a medium effect size (Cohen's *d* = 0.5) and a two‐sided *α* level of 0.05, the statistical power of the current sample was estimated to be only 35.1%. Therefore, these results indicate that the small sample size may lead to insufficient statistical power, which should be considered when interpreting the findings for all analyzed immune markers.

## 5 Conclusion

The adaptive immune response, especially T cell subsets, plays prominent role in NMOSD, whereas innate immune cells may contribute to the pathogenesis of AQP4‐IgG^−^ NMOSD. Certain innate immune response components (e.g., DCs and MDSCs) and surface molecules (e.g., CX3CR1 and CD80) may play a protective role in both AQP4‐IgG^+^ and AQP4‐IgG^−^ NMOSD groups and are correlated with certain clinical phenotypes.

## Author Contributions

Jingru Ren conducted Mendelian randomization analysis, collected clinical data, and drafted the manuscript. Jianchun Wang and Zhenyu Niu analyzed the data and created figures and tables. Jing Guo and Nan Zhang performed flow cytometry analysis. Ran Liu revised the manuscript. Hongjun Hao and Feng Gao provided technical support for flow cytometry. Zhaoxia Wang conceived and designed the study.

## Funding

The authors have nothing to report.

## Ethics Statement

This study has undergone ethical approval from the Peking University First Hospital Ethics Committee with the ethical approval number of 2019‐181 and has therefore been conducted in accordance with the ethical standards laid down in the 1964 Declaration of Helsinki and its subsequent amendments.

## Conflicts of Interest

The authors declare no conflicts of interest.

## Supporting information




**Table S1**: MR primary analysis (IVW) of 731 immune cells and NMOSD risk.
**Table S2**: MR results of the association between 731 immune cells and risk of total 215 NMOSD patients.
**Table S3**: MR results for the association between 731 immune cells and risk of 132 AQP4‐IgG^+^ NMOSD patients.
**Table S4**: MR results for the association between 731 immune cells and risk of 83 AQP4‐IgG^−^ NMOSD patients.
**Table S5**: Cochran's *Q* test and horizontal pleiotropy analysis results (MR‐PRESSO and MR‐Egger intercept test) for causal effects of prioritized immune cells on NMOSD.
**Table S6**: Leave‐one‐out analysis results (IVW) for causal effects of prioritized immune cells on NMOSD.
**Table S7**: Instrumental SNP variables for prioritized immune cells on NMOSD.
**Table S8**: MR Steiger analysis of the association between 731 immune cells and NMOSD risk.
**Table S9**: Treatment history of NMOSD patients in the flow cytometry validation cohort.
**Table S10**: The results of the correlation analysis between immune cell phenotypes and clinical data in this cohort.

## Data Availability

Data can be shared upon request and contact the corresponding author for details.
